# Dynamics of Microbial Populations Responsible for Biodegradation during the Full-Scale Treatment of Palm Oil Mill Effluent

**DOI:** 10.1264/jsme2.ME18104

**Published:** 2019-03-21

**Authors:** Diana Mohd-Nor, Norhayati Ramli, Siti Suhailah Sharuddin, Mohd Ali Hassan, Nurul Asyifah Mustapha, Hidayah Ariffin, Kenji Sakai, Yukihiro Tashiro, Yoshihito Shirai, Toshinari Maeda

**Affiliations:** 1 Department of Bioprocess Technology, Faculty of Biotechnology and Biomolecular Sciences, Universiti Putra Malaysia 43400, UPM Serdang, Selangor Malaysia; 2 Department of Biological Function and Engineering, Graduate School of Life Science and System Engineering, Kyushu Institute of Technology 2–4 Hibikino-cho, Wakamatsu-ku, Fukuoka 808–0196 Japan; 3 Laboratory of Biopolymer and Derivatives, Institute of Tropical Forestry and Forest Products (INTROP), Universiti Putra Malaysia 43400 UPM Serdang Malaysia; 4 Laboratory of Soil and Environmental Microbiology, Division of Systems Bioengineering, Department of Bioscience and Biotechnology, Faculty of Agriculture, Graduate School of Bioresources and Bioenvironmental Sciences, Kyushu University 744 Motooka, Nishi-ku, Fukuoka 819–0395 Japan

**Keywords:** bacterial community, methanogenic archaea, palm oil mill effluent, biodegradation, wastewater treatment

## Abstract

Despite efforts to address the composition of the microbial community during the anaerobic treatment of palm oil mill effluent (POME), its composition in relation to biodegradation in the full-scale treatment system has not yet been extensively examined. Therefore, a thorough analysis of bacterial and archaeal communities was performed in the present study using MiSeq sequencing at the different stages of the POME treatment, which comprised anaerobic as well as facultative anaerobic and aerobic processes, including the mixed raw effluent (MRE), mixing pond, holding tank, and final discharge phases. Based on the results obtained, the following biodegradation processes were suggested to occur at the different treatment stages: (1) *Lactobacillaceae* (35.9%) dominated the first stage, which contributed to high lactic acid production; (2) the higher population of *Clostridiaceae* in the mixing pond (47.7%) and *Prevotellaceae* in the holding tank (49.7%) promoted acetic acid production; (3) the aceticlastic methanogen *Methanosaetaceae* (0.6–0.8%) played a role in acetic acid degradation in the open digester and closed reactor for methane generation; (4) *Syntrophomonas* (21.5–29.2%) appeared to be involved in the degradation of fatty acids and acetic acid by syntrophic cooperation with the hydrogenotrophic methanogen, *Methanobacteriaceae* (0.6–1.3%); and (5) the phenols and alcohols detected in the early phases, but not in the final discharge phase, indicated the successful degradation of lignocellulosic materials. The present results contribute to a better understanding of the biodegradation mechanisms involved in the different stages of the full-scale treatment of POME.

In response to the increasing global demand for vegetable oils, the production of palm oil has been extensively promoted. However, the generation of a large amount of undesirable liquid waste, known as palm oil mill effluent (POME), has become a critical issue that needs to be resolved in order to satisfy the increasingly stringent environmental regulations regarding the final discharge (30). The presence of high levels of fat, as well as oil and grease, in the wastewater causes serious issues, not only in the receiving water, but also in the treatment plants and waste collection systems.

The use of a treatment system involving ponds for POME is the most popular method and has been adopted by more than 85% of palm oil mills in Malaysia. As shown in Fig. 1, hot mixed raw effluent (MRE), which is a mixture of wastewater produced through the oil manufacturing process from the sterilization and clarification stages, is placed in a mixing pond to be cooled down and is then moved to a holding tank. POME is subsequently treated in a closed reactor and open digester for the anaerobic process and then transferred to a facultative pond. POME is treated in the algae pond for the aerobic process and is finally released into a river. The use of anaerobic digestion reportedly meets effluent discharge standards. Moreover, the anaerobic system is a preferred choice due to its simple set-up and low cost of operation (7).

A previous study reported that pond and anaerobic treatment systems in mills use undefined microbial populations (27), which are responsible for the biodegradation of organic compounds and contaminants. The unknown mechanisms of action of degradative pathways may lead to digestion issues, resulting in a failure in the treatment system. Furthermore, POME influences microbial ecology; Sharuddin *et al*. (41) reported a shift in low nucleic acid (LNA) to high nucleic acid (HNA) bacterial cells in river water affected by the POME final discharge. Therefore, the issues associated with the treatment of POME cannot be resolved without understanding all of the biodegradative pathways involved.

The focus of recent studies has largely been on the microbial community structure of POME in various bioreactor configurations (34). However, less attention has been given to the bacterial and archaeal consortia involved in biodegradation at different stages of the full-scale treatment of POME. Further insights into the bacterial and archaeal communities involved as well as the key players catalyzing a complex series of biochemical reactions to reduce the polluting power of POME in the treatment system are needed. Substrates causing the inefficient treatment of POME have not yet been identified. Moreover, traditional monitoring approaches evaluated by assessing biochemical oxygen demand (BOD) and chemical oxygen demand (COD) removal efficiencies cannot be directly used to examine the relationship between the microbial biomass and any particulate organic matter present in the treatment system. Therefore, an assessment of the microbial community responsible for biodegradation in each stage of the treatment of POME is important for complementing current traditional monitoring approaches.

The recent development of high-throughput sequencing technologies, such as Illumina MiSeq, has contributed to the analysis of even low-abundance microorganisms, and, thus, may be used to more completely and accurately elucidate the compositions of microbial communities. In the present study, bacterial and archaeal diversities and compositions at different stages of the POME treatment were assessed using MiSeq sequencing, thereby providing detailed insights into the distribution of these populations and their roles in biodegradation processes. A comprehensive assessment of bacterial and methanogenic archaeal communities at the different stages will be beneficial for understanding the biodegradation mechanisms involved in the full-scale treatment of POME.

## Materials and Methods

### Sampling sites and sample collection

**Fig. 1:**
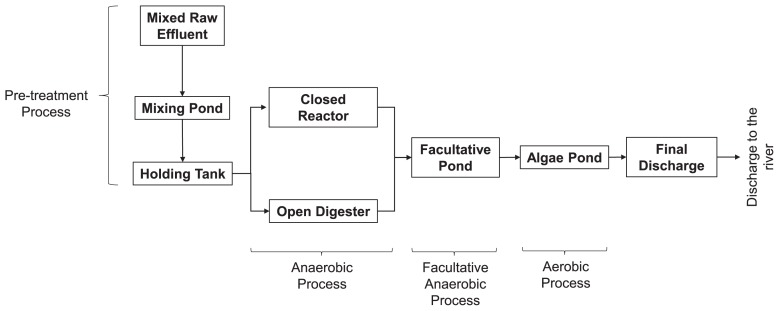
A process flow schematic of different stages in the full-scale treatment of palm oil mill effluent (POME) in a typical palm oil mill, comprising anaerobic and facultative anaerobic and aerobic processes, including mixed raw effluent (MRE), mixing pond, holding tank, and final discharge phases.

POME samples were collected three times a year in January, June, and December 2015 to examine variations caused by dry and rainy seasons in a tropical climate. Eight samples were collected from the different stages comprising the anaerobic as well as facultative anaerobic and aerobic processes, including the MRE, mixing pond, holding tank, and final discharge phases, in a typical POME treatment system. A simplified process flow diagram of the different stages of the POME treatment system is shown in Fig. 1. The characteristics of POME at each treatment stage are provided in Table 1.

Prior to the primary treatment, MRE, which is also known as raw POME, undergoes several pre-treatment stages that include the extraction of excess oil and grease, followed by a stabilization process that occurs in the mixing pond and holding tank. These processes are important for preventing the excessive formation of scum and also increase the production of oil (17). The primary treatment of POME occurs in the anaerobic process, either in the closed reactor and/or open digester. Open digesters are constructed of mild steel at different volumetric capacities. The closed reactor was designed by including a fixed or floating cover to capture the biogas produced, equipped with a gas collector, safety valves, and monitoring facilities. The open reactor and closed digester in the mill examined in the present study were constructed at a capacity of 1,800 m^3^.

A 2-L sample from each sampling point was collected in a pre-cleaned plastic container. Samples were collected from the collection pit at the end of each stage, except for MRE, which was taken from fresh raw POME. In bacterial and archaeal community analyses, each sample, except for the POME final discharge, was dispensed into a sterile Falcon tube and centrifuged at 14,000×*g* at 4°C for 10 min. After decanting the supernatant, the pellet was stored at −20°C prior to further analyses. A sample of the final discharge was filtered using Sterivex^TM^ filter units and stored at −20°C prior to further DNA extraction.

### Genomic DNA extraction, PCR, and Illumina MiSeq sequencing

Genomic DNA was extracted from approximately 2 g of sludge samples using the PowerMax^®^ Soil DNA Isolation Kit (Mo Bio Laboratories, Carlsbad, CA, USA) following the manufacturer’s instructions, except for the sample of the POME final discharge. Genomic DNA was extracted from the final discharge using the PowerWater^®^ Sterivex^TM^ DNA Isolation Kit (Mo Bio Laboratories, USA) according to the manufacturer’s instructions. DNA samples were stored at −20°C until further analyses. DNA quality was measured using a nano-spectrophotometer and visualized using 1.0% agarose gel electrophoresis.

Extracted DNA samples were amplified with a set of primers targeting the hypervariable V4–V5 region of the 16S rRNA gene. The forward primer was 515F (5′-GTGCCAGCMGCCGCGG-3′) and the reverse primer was 907R (5′-CCGTCAATTCMTTTRA GTTT-3′). Thermocycling steps were set as follows: an initial denaturation at 94°C for 3 min followed by 94°C for 45 s, 50°C for 60 s, and 72°C for 90 s of 35 cycles, and a final extension at 72°C for 10 min. PCR products were purified using the QIAquick Gel Extraction Kit (Qiagen, Valencia, CA, USA) according to the manufacturer’s instructions. Purified amplicons were assessed using the Qubit dsDNA HS Assay Kit (Life Technologies, Carlsbad, CA, USA). Amplicons were processed using the Nextera XT DNA Library Preparation Kit according to Illumina’s protocol (Illumina, San Diego, CA, USA). Amplicons were sequenced using Illumina MiSeq (V2 MiSeq reagent cartridge with 2×250-bp paired ends). The data obtained were demultiplexed and reads were then classified to different taxonomic levels.

### Bioinformatic analysis

Paired-end reads were assembled using PAired-eND Assembler for Illumina sequences (PANDAseq) (26) to improve the accuracy of the reads. A total of 1,134,163 16S rRNA gene sequence reads were processed by trimming using dynamic trim (12), followed by filtering and assigning operational taxonomic units (OTUs). OTUs were selected at 97% identity according to a de novo protocol using UCLUST (13). The reads from filtered OTUs were processed using the Quantitative Insights into Microbial Ecology (QIIME) program to construct a representative sequence for each OTU. Representative sequences were assigned at different taxonomic levels to the Greengenes databases using the USEARCH program (5). Alpha-diversity was then measured by the Shannon-Weaver index (H′) and Evenness (E′) using Paleontological Statistics (PAST) Software Package version 2.17c (16).

### Analytical methods

#### Physicochemical analyses

Temperature and pH values were recorded *in situ* using the portable meter. The assessment of BOD_5_ was conducted according to the procedure in Standard Method APHA 5210-B (3) and COD was measured using the reactor digestion method (HACH method 8000).

#### Organic acid measurement

The sample was centrifuged at 10,000×*g* for 15 min to remove the biomass. Organic acids were identified according to the analytical methods of NREL/TP-510-42623.43 (42). A 5-mL sample was pre-filtered using a 0.2-μm nylon membrane and injected into the high performance liquid chromatography (HPLC) system equipped with an ultraviolet (UV) detector (Shimadzu, Kyoto, Japan). Organic acids were separated on an Aminex HPX-87H column, 300×7.8 mm (Bio-Rad, Hercules, CA, USA) using 0.08 M sulphuric acid as the mobile phase at a flow rate of 0.6 mL min^−1^ and oven temperature of 50°C. Detection was performed using UV at 210 nm, while the peak of each acid was identified by referring the retention time obtained to that of standard compounds using a standard curve. All chemicals used in the preparation of standard solutions for HPLC analyses were of analytical grade.

#### Gas chromatography mass spectrometry (GCMS) analysis

The compositions of other lignocellulosic degradation products in samples were assessed using GCMS (Shimadzu) following the method of Chokwe *et al*. (9), with a detection limit for the peak area >2%. The liquid–liquid extraction pretreatment was conducted prior to the GCMS analysis using CH_2_Cl_2_ (chromatogram pure grade, Thermo Fisher Scientific, Waltham, MA, USA). A 20-mL pre-treated sample was freeze-dried prior to being dissolved in 2 mL of methanol. The sample was then filtered through a 0.22-μm nylon membrane before being analyzed. GCMS was equipped with a DB-5 column (Agilent, Santa Clara, CA, USA) with an internal diameter of 30 m×0.25 mm and film thickness of 0.25 μm. Conditions were set as follows: the initial oven temperature was held at 70°C for 2 min, increased at 20°C min^−1^ to 230°C, and then elevated to 270°C. Helium was used as the carrier gas at a flow rate of 1 mL min^−1^. The injector temperature was maintained at 250°C. A 1-μL sample was injected neat with a split ratio of 1:10. Mass spectra were recorded over the 50–650 amu range at 1 scan s^−1^ with an ionization energy of 70 eV and ion source temperature of 230°C. The compositions of samples were qualitatively identified through comparisons of their mass spectra in a library and published literature.

#### Sequence data

Raw sequence data have been deposited into the National Center for Biotechnology Information (NCBI) short reads archive database under accession number SRP108921.

## Results and Discussion

### Richness and diversity of bacterial and archaeal communities

The MiSeq analysis of 16S rRNA gene amplicons produced at least 82,687 effective sequences for each sample with an average length of 253 bp after removing low quality sequences. Sequences with ≥97% similarity were grouped into OTUs. The sequence number of each sample was normalized and 38,069–148,262 OTUs were generated using the QIIME platform. In the present study, the indices of H′ and E′ were used to measure the species richness and evenness of the distribution of species within a community, with higher values of indices being regarded as higher genetic diversity at a site. The richness and diversity of the bacterial and archaeal communities at the different stages of the POME treatment system are shown in Table 1. Based on H′ and E′, diversity increased throughout the treatment of POME.

Sharuddin *et al*. (40) previously reported that bacteria involved in biodegradation at the early phases may become excessively dominant, thereby lowering H′. Furthermore, the high concentration of organic matter readily available to most bacteria may contribute to increases in the bacterial population (21), which reduces H′ and E′ in the early phases of the POME treatment. A shift in the bacterial community during the treatment of municipal solid waste was previously reported with increased diversity over time based on the biodegradative ability of microorganisms during anaerobic digestion (6).

### Shift in the bacterial community involved in initial hydrolysis

**Fig. 2:**
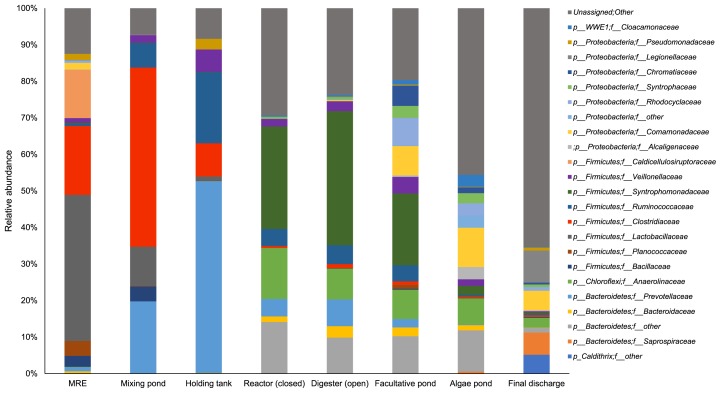
Relative abundance of bacterial and archaeal communities categorized at the taxonomic family level at different stages in the full-scale treatment of palm oil mill effluent (POME).

**Fig. 3:**
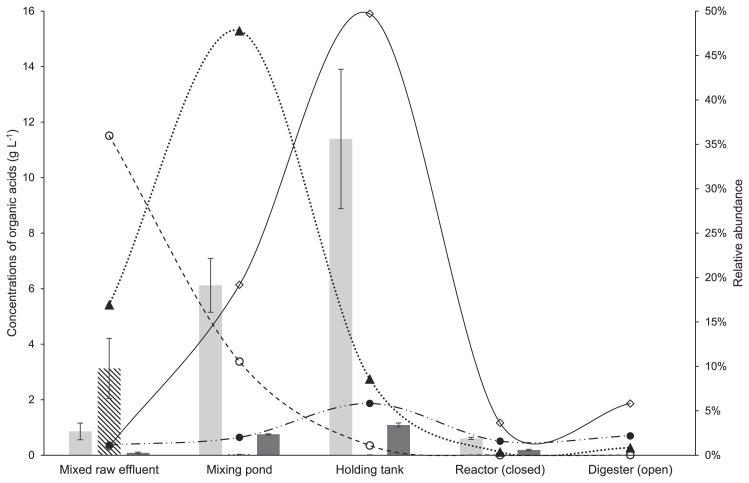
Relative abundance of *Lactobacillaceae* (○), *Veillonellaceae* (●), *Prevotellaceae* (⋄), and *Clostridiaceae* (▲) in relation to acetic ( 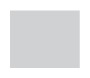), lactic ( 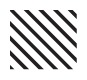), and propionic ( 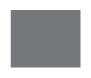) acid concentrations detected in mixed raw effluent (MRE), the mixing pond, holding tank, and closed reactor. No acids were detected in the open digester or the subsequent stages of the palm oil mill effluent (POME) treatment. Error bars represent standard deviations in experiments performed in triplicate.

The bacterial community at the family level shifted throughout the treatment of POME (Fig. 2). At the family level, 370 families was obtained and major sequences were classified into 23 families with ≥2% on average. MRE was suggested to contain microorganisms derived from palm fruit and lignocellulosic materials as well as various organic compounds generated during the process of oil palm production. *Lactobacillaceae* of the phylum *Firmicutes* showed the highest abundance in MRE (35.9%), in which it was suggested to contribute to higher lactic acid production (3.127±1 g L^−1^) (Fig. 3). The high proportion of *Lactobacillaceae* detected in MRE may have originated from remaining fiber because fresh raw POME contained a high concentration of suspended solids that mainly consisted of debris from the palm fruit mesocarp generated during the crude palm oil extraction process (23). Lactic acid bacteria were previously identified as the prominent community in the oil palm empty fruit bunch (OPEFB) due to the remaining oil content (4).

The present results showed the production of lactic acid by *Lactobacillaceae* as the earliest intermediate in MRE; however, as the reaction progressed, reductions were observed in the production of lactic acid, while increases were noted in that of propionic acid and acetic acid. Lactic acid was being converted to propionic acid, acetic acid, and CO_2_ by the family *Veillonellaceae* (11). The proportion of these bacteria was elevated in the mixing pond (2.0%) and holding tank (5.83%) (Fig. 2) in accordance with the increased concentrations of propionic acid and acetic acid (Fig. 3). However, the large increase observed in acetic acid to 6.121±1 g L^−1^ in the mixing pond was suggested to be produced by *Clostridiaceae* (47.8%) through the fermentation of cellulose and glucose. A recent study by Mustapha *et al*. (29) suggested that the large population of *Clostridia* in waste sewage sludge was involved in hydrolysis and acidogenesis. Meanwhile, further increments in the acetic acid concentration in the holding tank (11.393±3 g L^−1^) were suggested to be due to *Prevotellaceae*, a member of *Bacteroidetes*, which increased to 19.2% in the mixing pond and 49.7% in the holding tank. *Prevotellaceae* was previously described as a saccharolytic fermentative anaerobe involved in acidogenesis (24) with the ability to produce volatile fatty acids (VFAs), including acetic acid, as metabolic end products (38).

On the other hand, the high proportions of *Caldicellulosiruptoraceae* detected in MRE (11.9%) and *Ruminococcaceae* in the mixing pond (6.1%) and holding tank (17.3%) (Fig. 2) were suggested to play a role in initial cellulose hydrolysis. *Caldicellulosiruptor* sp. have been shown to utilize cellulose, cellobiose, xylan, and xylose through the actions of hydrolytic enzymes (33). Furthermore, *Ruminococcaceae* is known to consist of a number of cellulolytic and amylolytic species that exclusively appear in the biomass-derived cultures of anaerobic digestion (43).

### Biodegradation of lignocellulosic materials

The potential for lignocellulosic degradation was also noted during the treatment of POME. Alcohols detected in the early stages of the POME treatment (Table 2) have been proposed as the main degradation products of cellulose and hemicellulose (46). For example, the dominance of *Lactobacillaceae* in MRE and the mixing pond was suggested to be involved in the production of 1,3-propanediol and 2,3-butanediol. *Lactobacillus* has been reported to play a role in the bioconversion of glucose to 2,3-butanediol (8). With the presence of glycerol in POME (22), *Lactobacillus* was also proposed to function as a 1,3-propanediol producer from the co-fermentation of glucose and glycerol at the expense of ethanol and lactate (14). A higher population of *Clostridiaceae* in MRE and the holding tank was also suggested to be involved in the production of 1,3-propanediol. The utilization of lignocellulosic hydrolysates as co-substrates together with glycerol by *Clostridia* was found to enhance the production of 1,3-propanediol (45). The disappearance of these compounds in the subsequent stages may be due to major reductions in the populations of *Lactobacillaceae* and *Clostridiaceae*.

Phenolic compounds were detected as major degradation products in MRE (Table 2), and this was suggested to be due to the presence of lignin in lignocellulosic materials. Sharip *et al*. (39) previously characterized the oil palm mesocarp fiber superheated steam condensate and identified phenolics as the major compounds potentially contributing to lignin degradation. The high temperature and acidic pH of raw POME (20) has been proposed to assist in the degradation of lignin. According to Álvarez *et al*. (1), the treatment of lignocellulosic material (sawdust) with acid may modify its chemical composition. Moreover, the hydrothermal pretreatment of the oil palm biomass may partially remove hemicellulose and modify the lignin structure, resulting in changes to the cellulose-hemicellulose-lignin matrix (47).

The presence of *Comamonadaceae* in MRE (1.3%) has been suggested to contribute to the recycling of the plant-derived carbons of aromatic compounds, including phenols (32). *Alcaligenaceae* in MRE (0.1%) were shown to have potential in the degradation of phenolics (35) and aromatic (32) compounds. However, *Bacillaceae* (3.4%) and *Pseudomonadaceae* (2.7%), the populations of which increased in the holding tank, have been proposed to play a role in the degradation of aromatic compounds (37). The present results indicated that phenols and alcohols present in MRE were degraded throughout the POME treatment. These compounds were mostly detected in MRE and the mixing pond, and not in the final discharge, indicating the successful degradation of lignocellulosic materials in the POME treatment system.

### Shift in bacterial and archaeal communities involved in methanogenesis

A very large shift in the bacterial community was recorded as the anaerobic treatments in the closed reactor and open digester started (Fig. 2). The shift in methanogenic populations in the closed reactor and open digester was suggested to influence the generation of methane, as reported previously (10). The concentrations of VFAs, including acetic acid, have been shown to correlate with the population of methanogens in an anaerobic system. In the present study, increases in the aceticlastic methanogen *Methanosaetaceae* in the closed reactor (0.8%) and open digester (0.6%) were suggested to play a role in degrading acetate for the generation of methane, which was supported by the large reduction observed in acetic acid in the reactor (Fig. 3).

The large increases in *Syntrophomonas* in the closed reactor (21.5%) and open digester (29.2%) may also function as acetate-oxidizing bacteria if the hydrogen/CO_2_ produced are subsequently utilized by hydrogenotrophic methanogens (19). Furthermore, the co-culture of the hydrogenotrophic methanogen *Methanobacteriaceae* (0.6–1.3%) with *Syntrophomonas* in the closed reactor and open digester may contribute to the β-oxidation of fatty acids. In a previous study by Hatamoto *et al*. (18), the co-existence of the hydrogenotrophic methanogen *Methanospirillum hungatei* with *Syntrophomonas palmitatica* sp. nov. isolated from granular sludge in a mesophilic upflow anaerobic sludge blanket reactor for the treatment of POME was shown to oxidize straight-chain saturated fatty acids, including palmitate with carbon chain lengths of C4–C18. Long-chain fatty acids, particularly palmitate, were previously detected in POME (15). This co-culture was shown to play a role in the β-oxidation of fatty acids, with fatty acids with an odd number of carbon atoms being converted into propionate and methane, while acetate and methane were produced from fatty acids with an even number of carbon atoms. The presence of hydrogenotrophic methanogens and syntrophic bacteria is important for achieving maximum COD removal and optimum biogas production (2).

**Fig. 4:**
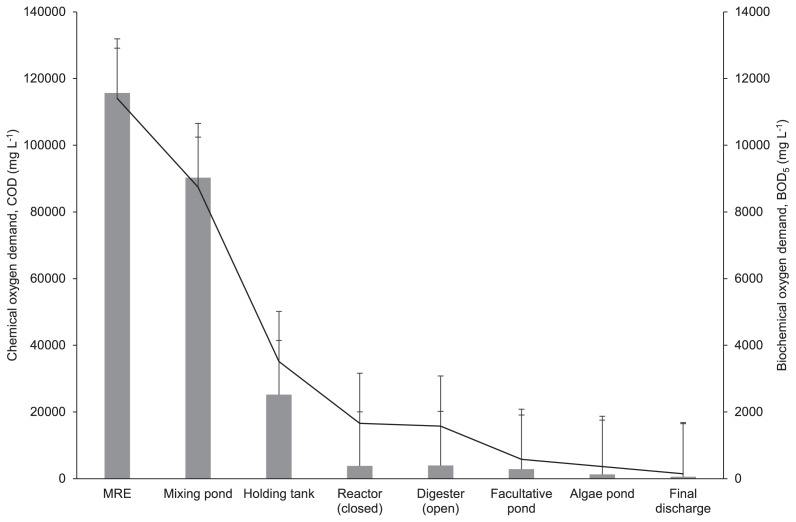
Changes in biochemical oxygen demand, BOD_5_ ( 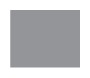), and chemical oxygen demand, COD (–), throughout different stages in the palm oil mill effluent (POME) treatment. Error bars represent standard deviations in experiments performed in triplicate.

**Fig. 5:**
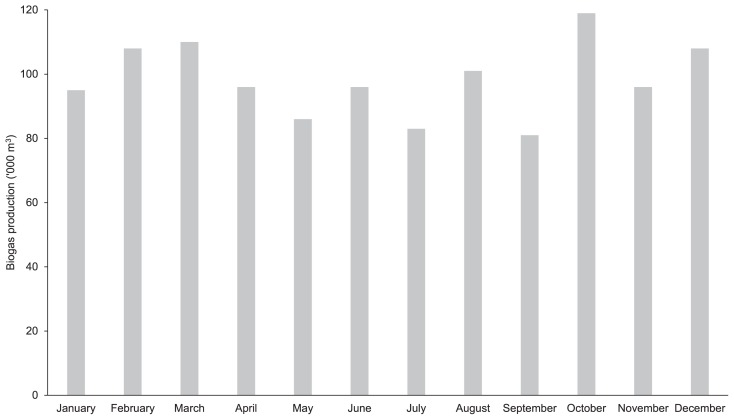
Monthly production of biogas in commercial scale reactors (four units) with the capacity of 1,800 m^3^ each in the palm oil mill examined in 2015 (data provided by the palm oil mill).

The concentrations of BOD_5_ and COD, as well as total biogas production, were measured in the present study in order to demonstrate the functionality of the anaerobic treatment. Large reductions in the concentrations of BOD_5_ were detected in the closed reactor (85.4%) and open digester (86.2%). According to Satyawali *et al*. (36), more than 80% of BOD may be removed from an anaerobic treatment with the potential for energy recovery in the form of biogas. The COD removal rate has also been shown to affect the maximum production of methane (25), with more than 50% of COD being converted to biogas (44). COD removal was recorded with an 84.8% reduction in the closed reactor and 84.3% reduction in the open digester (Fig. 4), indicating the satisfactory performance of anaerobic processes. In the present study, biogas production was also measured from the commercial scale of closed reactors (1,800 m^3^ each) with a total of 1,178,620 m^3^ year^−1^ (Fig. 5), demonstrating that the anaerobic treatment functioned well.

### Shift in the bacterial community in later stages of the POME treatment

*Comamonadaceae* and *Alcaligenaceae* emerged in the facultative pond, algae pond, and final discharge (Fig. 2), and have been suggested to function in the degradation of certain compounds resulting from ultraviolet (UV) irradiation. The selected group of compounds present in the aquatic environment may be degraded by UV photolysis and UV/H_2_O_2_ oxidation (31). *Alcaligenaceae* were previously reported to be present in a number of POME final discharges treated by different biotreatment processes, and, thus, were proposed as one of the reliable bioindicators of river water contamination due to POME final discharges (28). Meanwhile, the abundance of *Syntrophomonas* in the facultative pond (15.0%) may have been carried over from the upstream anaerobic process.

**Fig. 6:**
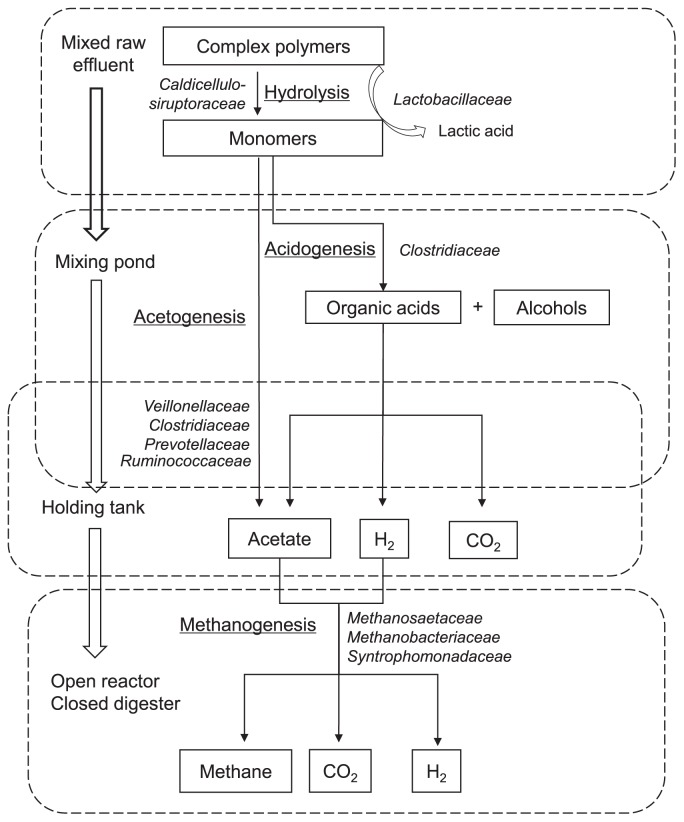
Schematic flow process diagram of biodegradation at different stages during the treatment of palm oil mill effluent (POME) catalyzed by dominant bacteria and archaea populations for the generation of methane as the end-product.

The present results indicated that bacterial and methanogenic archaeal communities shifted due to biodegradation at the different stages of the full-scale treatment of POME. Biodegradation by dominant bacteria from hydrolysis to methanogenesis is shown in Fig. 6. The results of the present study provide insights into the importance of bacterial and methanogenic archaeal communities and their balanced populations in catalyzing the biodegradation of a number of compounds throughout the POME treatment. The information obtained herein is an important first step in our understanding of the mechanisms involved in the different treatment stages of POME.

## Figures and Tables

**Table 1:** Characteristics and alpha-diversity analyses of the bacterial and archaeal communities at different stages in the palm oil mill effluent (POME) treatment

POME treatment processes/Parameters	Mixed raw effluent	Mixing pond	Holding tank	Anaerobic		Facultative anaerobic pond	Aerobic (algae) pond	Final discharge

				Closed reactor	Open digester			
*Characteristics*								
pH	4.58±0.2	4.42±0.3	4.39±0.2	7.08±0.0	7.07±0.0	8.08±0.1	8.03±0.0	8.04±0.0
Temperature (°C)	65.57±0.8	43.63±0.7	41.62±1.0	40.59±2.8	37.89±1.3	32.92±1.4	32.95±1.7	31.91±0.7
Retention time (d)	0	1–2	1	20	20	20	14	0
*Alpha-diversity analyses*								
No. of sequences^a^	115880	140354	196849	187554	82687	95693	114515	200631
OTU^b^	93854	93681	148262	101477	38069	55923	63442	121368
Shannon-Weaver index (H′)	0.869	0.800	1.119	1.908	1.725	1.933	1.929	2.272
Evenness (E′)	0.050	0.077	0.073	0.120	0.115	0.111	0.109	0.145

Notes:

a Detected sequence number;

b Detected OTU number

**Table 2:** Presence of alcohols and phenolics as main degradation compounds from lignocellulosic materials at different stages during the full-scale treatment of palm oil mill effluent (POME)

Name of Compounds	MRE^a^	MP^b^	HT^c^	CR^d^	OD^e^	FP^f^	AP^g^	FD^h^
*Alcohols*								
1,3-Propanediol	√	√	√	√	√	—	—	—
2,3-Butanediol	√	√	—	—	—	—	—	—
Benzyl alcohol	√	—	—	—	—	—	—	—
Phenylethyl alcohol	√	—	—	—	—	—	—	—
3-Hexanol, 4-methyl	√	√	—	—	—	—	—	—
Resorcinol	√	—	—	—	—	—	—	—
3-Pyridinol	√	—	—	—	—	—	—	—
Triethylene glycol	√	—	—	—	—	—	—	—
*Phenolics*								
1,2-Benzenediol	√	√	√	—	—	—	—	—
Phenol, 3,4,5-trimethoxy-	√	—	—	—	—	—	—	—
Phenol, 2-methoxy-	√	—	—	—	—	—	—	—
2-Methoxy-4-vinylphenol	√	—	—	—	—	—	—	—
Phenol, 2,6-dimethoxy-	√	—	—	—	—	—	—	—
3-Pyridinol	√	—	—	—	—	—	—	—
Phenol, 4-(2-propenyl)-	√	—	—	—	—	—	—	—
Tyrosol	√	—	—	—	—	—	—	—
Homovanillic acid	√	—	—	—	—	—	—	—
Coumaran	√	—	—	—	—	—	—	—

Notes:

a mixed raw effluent,

b mixing pond,

c holding tank,

d closed reactor,

e open digester,

f facultative pond,

g algae pond,

h final discharge
